# A protocol for a systematic literature review of economic evaluation studies of interventions to address antimicrobial resistance

**DOI:** 10.1186/s13643-021-01794-3

**Published:** 2021-09-07

**Authors:** Aparna Ananthakrishnan, Chris Painter, Yot Teerawattananon

**Affiliations:** 1 Health Intervention and Technology Assessment Program (HITAP), Ministry of Public Health, Tiwanon Road, Muang District, 11000 Nonthaburi, Thailand; 2 Overseas Development Institute, London, UK

**Keywords:** AMR, Cost-effectiveness, Economic evaluation, Interventions, Cost-benefit analysis

## Abstract

**Background:**

Antimicrobial resistance (AMR) is accelerated by the widespread and often indiscriminate use of antimicrobials in humans, animals, and the environment. In 2015, the World Health Organization recognised AMR as one of the top ten global health threats, due to its potential to neutralise humanity’s advancements in western medicine by enabling the emergence of new strains of existing pathogens, many of which have no available treatments. Over the past decade, several countries, including those in low- and middle-income contexts, have started implementing interventions to tackle AMR. However, economic evidence regarding the cost-effectiveness of these interventions remains weak. To address this evidence gap, we will conduct a systematic literature review to provide a comprehensive summary on the value for money of different AMR interventions.

**Methods:**

We aim to conduct a systematic literature review of all available economic evaluations on interventions addressing AMR and will provide a narrative synthesis of our findings. Systematic searches for relevant studies will be performed across all suitable databases as well as in grey literature sources such as unpublished studies, reports, and other relevant documents. All economic evaluation studies will be included as long as they report an economic outcome and have stated that the analysed intervention will reduce antimicrobial resistance or antimicrobial use in the abstract. Those studies reporting clinical endpoints alone will be excluded. Selection for final inclusion and data extraction will be performed by two independent reviewers.

**Discussion:**

The review will be one of the first of its kind, and the most recent, to systematically review literature on the cost-effectiveness of AMR interventions, an important evidence gap in the economics of AMR. The findings will enable policy and decision-makers, particularly in resource-constrained settings, to better use available resources when selecting interventions to address AMR burdens,

**Systematic review registration:**

PROSPERO CRD42020190310

**Supplementary Information:**

The online version contains supplementary material available at 10.1186/s13643-021-01794-3.

## Introduction

In 2019, the World Health Organization (WHO) listed antimicrobial resistance (AMR) as one of the top ten threats to global health, due to the catastrophic impact it has the potential to cause [1]. The Review on Antimicrobial Resistance estimated that inaction in addressing AMR could result in an estimated 10 million deaths per year by 2050 and yield a much greater economic impact than that of the 2008–2009 financial crisis [2]. With the onset of the current COVID-19 pandemic, there has been a growing urgency towards further investigating issues of resistance in the light of the spread of the novel infectious disease.

Antimicrobial resistance (AMR) occurs when pathogens (bacteria, viruses, fungi and parasites) develop a resistance or tolerance to the medicines that are used to combat these microorganisms, such that these treatments are no longer effective [3]. Although AMR is a natural phenomenon, the speed at which it occurs is impacted by how much exposure pathogens have to treatments. There have been numerous publicised cases of pathogens developing AMR, including methicillin-resistant *Staphylococcus aureus* (MRSA), drug-resistant tuberculosis and antibiotic-resistant gonorrhoea [4, 5], which are all far more harmful strains than the original pathogens.

AMR has been increasing in low-, middle- and high-income countries around the world in recent years, and this trend is expected to continue [5–7]. Klein et al. [8] conducted a trend analysis on antibiotic consumption between 2005 and 2015 in 76 countries. The results indicate that between this time period, antibiotic consumption rose globally by 65% (measured by defined daily doses [DDD], a standard drug intake metric), primarily driven by increases in consumption in low-and middle-income countries (LMICs); estimates suggest a 77% increase in antibiotic consumption rate per 1000 inhabitants in these regions. Projecting global consumption patterns in 2030 with no policy interventions, the study estimated a 200% increase. This increase has been principally driven by increase in global demand for antibiotics, which are overused and, in many cases, misused (e.g. the use of antibiotics for common viral infections, like the flu, in humans and as growth promoters in farm animals) and compounded by falling investment in the development of new antimicrobial agents [9]. The indiscriminate use of antibiotics in the animal farming sector, where healthy animals are given antibiotics as a precaution, is just part of what makes the livestock industry responsible for an estimated 70–80% of the global total of antibiotic consumption [10]. However, antibiotics have undoubtedly been overused by humans and in agriculture practices (both livestock and crop farming) [1, 11]. These drivers respond to economic incentives as seen in physician-patient relationships, farming practices for greater yields as well as other environmental factors. While these drivers are not unique to LMICs, their impacts can be felt more severely in these regions due to a lack of regulation and other socio-economic factors.

Interventions need to consider the multisectoral nature of AMR if it is to be controlled as a public health threat, using a One Health approach which recognises the links between antimicrobial use in humans, animals and the environment [11]. The WHO’s 2015 Global Action Plan on AMR identified several key methods for reducing AMR as a threat, including through (1) optimisation of the use of antimicrobials in both human and animal health; (2) reducing infections, through effective sanitation, hygiene and other infection prevention measures; and (3) sustainable investment in the development of new antimicrobials, diagnostic tools and other interventions [12]. Using this framework, over the last few years, many countries have attempted to improve their data collection systems and formulate interventions and policy measures to address AMR, primarily through their own domestic policies but also by contributing to the global policy landscape. National AMR Plans, in line with the WHO framework detailing country-specific interventions, have been drawn up and operationalised in many countries [12]. However, many of these initiatives have little to no evidence concerning their relative costs and benefits; an issue of significant importance, particularly for resource-constrained settings such as LMICs which face multiple demands on their budgets.

The Global Action Plan on AMR also emphasises the need to bridge the existing gap in economic research to support improved AMR awareness (objective 1) as well as the urgency of ensuring an economic-evidence based use of interventions to feed into the development of a financial case for investment in AMR diagnostics and treatments (objective 5) [11]. LMIC governments face many competing interests for new health investments, and there has been an increasing focus on using economic evaluation and health technology assessment (HTA) to inform resource allocation decisions in healthcare and maximise the value for money of the health system overall [13]. Economic analysis on interventions to address AMR is a necessary part of the required evidence base for justifying government expenditure and investment in interventions of this type and therefore is a key step in AMR prevention and control. LMICs have developed growing capacity to conduct health economic evaluations in recent years [14], yet the most recent systematic review on the cost-effectiveness of measures to contain the occurrence of AMR dates back to 2002 [15]. This study highlighted that inadequate evidence was available on this subject of economics for AMR interventions and more investigation was required. Since then, little has been done to improve available evidence; however, there have been reviews focussed solely on the impact of antimicrobial stewardship programmes in hospitals or the economic burden of antimicrobial resistance [16, 17].

In order to bridge this important evidence gap and contribute to the objectives outlined by the WHO Global Action Plan on the important evidence needs in the realm of economics of AMR, this systematic review aims to detail data from economic evaluations regarding the value-for- money of these interventions as a step towards optimising resource use in tackling AMR. In specific, this review will answer the following questions:

- Objective 1: What interventions to address antimicrobial resistance have been the subject of an economic evaluation?
- Objective 2: In what types of setting (e.g. high-income, low-income, regions etc.) have these economic evaluations been focused?
- Objective 3: Which interventions have been estimated to be cost-effective, and has this result been replicated in other settings/contexts?
- Objective 4: What economic evaluation methods or techniques have been used to evaluate these interventions?
- Objective 5: What kind of data has been used in conducting economic evaluations for these interventions? What is the quality of this data?

## Methods

This protocol has been developed using the “The Preferred Reporting Items for Systematic Reviews and Meta-Analyses Protocols” (PRISMA-P) [15].

### Inclusion criteria

We will include both trial-based and model-based economic evaluations published in the English language in the literature review, published from the year 2000 onwards. Any category of full economic evaluation will be included, which is defined as a study that consider the costs and effects of two or more interventions: cost-effectiveness analysis (CEA), cost-benefit analysis (CBA), cost-utility analysis (CUA), cost-minimisation analysis (CMA) and budget impact analyses [18, 19].

### Exclusion criteria

Studies on interventions to reduce AMR which report only clinical endpoints and do not investigate any economic outcomes will be excluded from the review. Reviews, editorials, commentaries and methodological articles will also be excluded. Citations in any systematic reviews will be reviewed to examine relevant studies for inclusion; however, the reviews themselves will be excluded from this literature review. Studies conducted before the year 2000 will be excluded, as the most recent systematic review of this nature conducted searches up to this date [15].

### Type of populations

The study populations will include humans, animals and the environment, consistent with the One Health approach of addressing AMR [11].

### Type of interventions

The types of intervention that we will be including in the review are outlined below; these categories have been adapted from an evidence synthesis of human AMR community and primary care interventions produced by the Government of Canada [20]. As this is an incipient field of global research, this list is not exhaustive, but only provides some examples on the diverse interventions that countries have been engaged in.

- National and international government policies and legislations, such as National Action Plans introduced to address the issue of AMR, partnerships and collaborations such as the WHO Global Action Plan on AMR (GAP-AMR) or the tripartite collaborations between WHO, FAO and OIE
- Healthcare processes and guidelines in hospital settings and others such as laboratories; agricultural and livestock-based settings
- Antimicrobial stewardship programmes at hospitals and in other settings like clinics, communities and agricultural farms
- Pharmaceutical interventions used with the aim of reducing AMR
- Medical technologies such as medical and in vitro diagnostics for surveillance, monitoring bacterial response to medicines, control of hospital-acquired infections
- Awareness generation activities such as media campaigns and advocacy efforts to reduce/control the unnecessary use of antimicrobials

To be included in review, the abstract of the article must state that the intervention has an effect of reducing antimicrobial resistance in some way, either through controlling the spread of resistant microbials, eradicating resistant microbials or reducing inappropriate antimicrobial use.

### Type of outcome measures

The types of outcome measures that this review will record include any cost-benefit measurement such as incremental cost-effectiveness ratio (ICER), incremental cost per quality-adjusted life year (QALY), incremental cost per disability-adjusted life year (DALY), incremental cost-benefit ratio, net monetary benefit (NMB), incremental net benefit (INB), net health benefit (NHB), costs avoided, net costs, cost-consequence measures and budget impact. Our review will also include outcomes that are specific to the AMR context, such as incremental cost per resistant-infection avoided. This also applies to veterinary or animal settings, which may be more likely to use cost-benefit analyses and report all outcomes as costs. We note that there are limited publications of economic evaluations for environmental interventions, and routine methods and outcomes for the evaluation of natural environment interventions have not yet been established [21, 22]. Furthermore, this review will analyse the types of settings (countries or regions, country income-levels, farms, pharmacies or hospitals, types of hospitals (primary, community or tertiary) that these analyses were focussed in.

### Search methods

We will use a combination of published and grey literature sources to inform this review. We will review global databases in the domains of health economics and public health, as outlined here.

Electronic searches will be conducted of the following databases:

- MEDLINE (Ovid)
- EMBASE (embase.com)
- Cochrane Library
- Web of Science
- Tufts Cost Effectiveness Analysis (CEA) Registry and Global Health (GH) CEA Registry
- Centre for Reviews and Dissemination’s National Health Service Economic Evaluation Database (NHS-EED)

Grey literature searches will also be conducted, including using international conference databases for the following conferences: Health Technology Assessment International (HTAi), International Society of Pharmacoeconomics and Outcomes Research (ISPOR) and International Health Economics Association (iHEA). Hand searches of the bibliographies of any included studies will be performed to identify any overlooked publications of relevance.

### Search strategy

A search strategy that prioritises sensitivity will be developed, due to the expectation that relatively few economic evaluations focussing on AMR have been conducted. The search strategy will be created following the guidance available from the Centre for Reviews and Dissemination’s Guidance for Undertaking Reviews in Healthcare and will use appropriate keywords (MeSH terms) associated with study objectives (model type) [23]; the preliminary search strategy for the MEDLINE (Ovid) can be found in the supplementary materials. The Peer Review of Electronic Search Strategies (PRESS) checklist for systematic reviews will also be considered when designing the search strategy [24].

### Study selection and data extraction

Data will be exported to Covidence based on the inclusion and exclusion criteria of this review.

The inclusion of relevant systematic reviews will be conducted according to a two-step process:

1. Two reviewers will independently screen titles and abstracts of all papers initially retrieved. In case of disagreements, consensus will be sought through discussion.
2. Full text screening of selected systematic reviews will be conducted by two independent reviewers. In case of disagreements, consensus will be sought through discussion.

References will be imported into Covidence Systematic Review software for selection and coding. Data that will be extracted from the systematic reviews includes:

- Country analysed in the study
- Study setting (e.g. hospitals)
- The first and corresponding authors’ affiliation
- Year of publication
- Type of publication (and journal if, if applicable)
- Funders
- Intervention
- Comparator
- Perspective
- Time horizon
- Discounting
- Methodology (modelling approach)
- Type of model
- Types of costs and base year for costing data
- Sources of cost data
- Types of outcome measures
- Results (e.g. ICER value, incremental costs, incremental effectiveness outcomes)
- Cost-effectiveness thresholds used
- Type of uncertainty analysis conducted and key sources of uncertainty in the model, if identified
- Conclusion (e.g. whether an intervention is deemed worthwhile/cost-effective/good value for money)
- Examined clinical endpoints

Three researchers will extract data. One researcher will extract the data and another researcher will be responsible for checking the accuracy of the extracted data. In case of disagreements, a fourth researcher will be consulted to resolve the conflict. If there are missing data, the study authors will be contacted to retrieve these.

### Quality assessment

The quality assessment will be multi-faceted and will correspond to different objectives of the systematic review. Objectives 1, 3–5: An effort will be made to assess the quality of economic evidence for outcomes related to resource use, as recommended by the Grading of Recommendations Assessment, Development, and Evaluation (GRADE) approach [25, 26]. This quality assessment has five domains: (1) risk of bias/study limitations, (2) inconsistency, (3) indirectness, (4) imprecision and (5) publication bias. A GRADE evidence profile and summary of findings table will be developed using GRADE Pro Software [26]. Objective 4: The risk of bias/methodological quality of included publications will be assessed independently by two authors using the following tools:

- Trial-based economic evaluation studies: Cochrane Collaboration’s tool for assessing risk of bias [27], Consensus Health Economic Criteria (CHEC) Criteria list [28], and the Drummond Checklist [29]
- Model-based economic evaluation studies: Philips checklist [30]

Objective 5: The quality of the data used in included economic evaluation studies will be assessed using an adapted framework for the hierarchy of evidence scoring system detailed in Cooper et al. 2005 [31].

### Data analysis and synthesis

We will conduct a narrative synthesis of the economic evaluations to report our findings [32]. The objective of this review will not be to estimate a cost-effectiveness measure of a single AMR intervention, but rather to provide a descriptive synthesis of the economic evidence available on the value-for-money of the different interventions that have been implemented globally. This method of synthesising the data seems to be most applicable to our study as we anticipate the inclusion of a broad variety of study objectives and methods that fit within our study criteria [33]. In addition to the structured narrative synthesis which will answer all 5 objectives of the study, we also propose using tabulations and graphical representations where applicable to showcase the findings for each of our objectives.

For objectives 1–4, we will include a condensed form of the data extraction tables which will summarise and report on the different settings (e.g. high income, low income) and regions (e.g. continents, countries, regional blocs) in which these economic evaluations have been conducted, the methodology used (e.g. cost effectiveness, cost utility etc.), the cost-effectiveness conclusion, the inclusion of sensitivity analysis and parameters used for the same, the populations targeted by the interventions (e.g. farm animals, companion animals, humans), the type of interventions analysed, the perspective used and the types of funders for the economic evaluations. In specific, for objective 2, we will also incorporate a geographical depiction such as a world map to present the regional distribution of the included studies. If there are sufficient included studies, we also propose stratifying the results by pre-specified categories of intervention, such as screening, primary care, hospital treatment, policy decisions and educational programmes. Stratified results will also be reported in tabular formats to facilitate a comprehensive understanding of available data and a quick overview into the different sub-groups by which the data has been classified. Additionally, this review will aim to identify if there exists a consensus in the literature on the cost effectiveness of specific AMR interventions and also make note of any methodological considerations in the studies as well as pertinent study characteristics to inform future research. We will also summarise the types of data used in these economic evaluations and their respective quality, reported in a tabular format. The synthesis of the extracted data will be further informed by suggestions from the ISPOR Good Practices Task Force Report and will be determined once the data have been extracted from the included studies [33].

## Discussion

To our knowledge, no systematic reviews of economic evaluations of any intervention to address AMR have been conducted recently; the earliest attempt of a similar study dates back to 2002 after which none have been published. Though we do not anticipate that a large number of studies will be identified, we believe it is essential to understand the current landscape of economic evaluation literature on AMR interventions. We also recognise that the English language requirement in our inclusion criteria will potentially limit the number of relevant studies, though regretfully we are unable to review articles in other languages. However, this research will still be important in highlighting critical evidence gaps in the growing literature on the economics of AMR, (1) synthesising current economic evidence on AMR interventions (2), providing direction for future research priorities within economic evaluations for AMR interventions by identifying information and methodological gaps such as the use of discounting, time horizons and perspectives as well as quality of data on utilities and outcomes and (3) build capacity towards increased evidence-informed policy making, especially for countries in resource-constrained settings. This review may also help guide other evidence generation activities (such as the need for better evidence regarding the effectiveness of interventions or costing information, both in general and in specific contexts such as LMICs). Most importantly, this review will re-emphasise that more research is required to bolster economic evidence on AMR interventions, especially as we battle a virulent pandemic outbreak and reflect on broader issues of global health security for our future.

## Supplementary Information

**Additional file 1.** Preliminary search strategy for the MEDLINE (Ovid).

## Data Availability

Not applicable.
