# Supplementary material for: A protocol for a systematic literature review of economic evaluation studies of interventions to address antimicrobial resistance
Source: Syst Rev. 2021 Sep 7;10:242. doi: 10.1186/s13643-021-01794-3 (PMC8421239; doi:10.1186/s13643-021-01794-3)
Supplement: Supplementary file 1 — Additional file 1. Preliminary search strategy for the MEDLINE (Ovid). [file 13643_2021_1794_MOESM1_ESM.pdf]

MEDLINE Search terms for AMR EE SLR

1.	exp Economic Development/ or exp Models, Economic/ or exp Economic Competition/ or exp Inflation, Economic/ or exp Economic Recession/ or exp "fees and charges"/ or exp Budgets/
2.	exp "costs and cost analysis"
3.	(economic* or pharmacoeconomic* or "economic analysis" or "economic evaluation" or "economic stud*" or "economic modelling" or price* or pricing).mp.
4.	("cost benefit" or "cost effective" or "cost analysis" or "cost minimisation" or "cost utility analysis" or "budget impact").mp.
5.	(cost or "cost description" or "cost saving" or "cost shar*" or "cost allocation").mp.
6.	("deductibles and coinsurance" or (fiscal or funding or financial or finance)).tw.
7.	(health?care adj cost$).mp.
8.	((low adj cost) or (high adj cost)).mp.
9.	(cost adj estimate*) or ((cost adj variable*) or (unit adj cost*).mp.
**10.**	**(1 or 2 or 3 or 4 or 5 or 6 or 7 or 8 or 9)**
11.	exp "Drug Resistance, Microbial"/ or exp "Antibiotic Resistance"/ or exp "Antibiotic Resistance, Microbial"/or exp "Antimicrobial Drug Resistance"
12.	("antimicrobial drug resistan*" or "drug resistan*" or "antibiotic resistan*" or "antimicrobial resistan*" or "multi-drug resistan*").mp
13.	("antimicrobial drug resistan*" or "antibiotic resistan*" or "antimicrobial resistan*").tw
**14.**	**(11 or 12 or 13)**
**15.**	**(10 and 14)**
**16.**	**limit 15 to yr="2000 -Current"**
**17.**	**limit 16 to english language**
